# The Deformation Monitoring Capability of Fucheng-1 Time-Series InSAR

**DOI:** 10.3390/s24237604

**Published:** 2024-11-28

**Authors:** Zhouhang Wu, Wenjun Zhang, Jialun Cai, Hongyao Xiang, Jing Fan, Xiaomeng Wang

**Affiliations:** 1 School of Environment and Resources, Southwest University of Science and Technology, Mianyang 621010, China; shouyeren@mails.swust.edu.cn (Z.W.); fanjing@mails.swust.edu.cn (J.F.); wangxiaomeng@mails.swust.edu.cn (X.W.); 2 Mianyang Science and Technology City Division, The National Remote Sensing Center of China, Mianyang 621010, China; 3 School of Civil Engineering and Architecture, Southwest University of Science and Technology, Mianyang 621010, China; xianghongyao@mails.swust.edu.cn

**Keywords:** Fucheng-1 (FC-1), PSI, DSI, subsidence of land surface, interferometric synthetic aperture radar (InSAR)

## Abstract

The Fucheng-1 (FC-1) satellite has successfully transitioned from its initial operational phase and is now undergoing a detailed performance assessment for time-series deformation monitoring. This study evaluates the surface deformation monitoring capabilities of the newly launched FC-1 satellite using the interferometric synthetic aperture radar (InSAR) technique, particularly in urban applications. By analyzing the observation data from 20 FC-1 scenes and 20 Sentinel-1 scenes, deformation velocity maps of a university in Mianyang city were obtained using persistent scatterer interferometry (PSI) and distributed scatterer interferometry (DSI) techniques. The results show that thanks to the high resolution of 3 × 3 m of the FC-1 satellite, significantly more PS points and DS points were detected than those detected by Sentinel-1, by 13.4 times and 17.9 times, respectively. The distribution of the major deformation areas detected by both satellites in the velocity maps is generally consistent. FC-1 performs better than Sentinel-1 in monitoring densely structured and vegetation-covered areas. Its deformation monitoring capability at the millimeter level was further validated through comparison with leveling measurements, with average errors and root mean square errors of 1.761 mm and 2.172 mm, respectively. Its high-resolution and high-precision interferometry capabilities make it particularly promising in the commercial remote sensing market.

## 1. Introduction

Ground deformation refers to the process of rising or sinking of the Earth’s surface caused by geological changes or environmental impacts [1,2]. Ground subsidence may follow long periods, and the sinking rate and cumulative effects may be significant. Traditional geodetic surveying and global navigation satellite systems (GNSSs) have long been used to map the current surface deformation [3,4]. However, due to the labor-intensive nature of these methods, an increasing number of scholars are using InSAR (interferometric synthetic aperture radar) technology to monitor surface deformation [5,6]; this is a remote sensing technology that measures ground deformation using radar satellites, and GNSSs have also become an important means of verifying the accuracy of InSAR results [7]. Since Graham [8] proposed using InSAR technology to monitor small-scale surface deformations in 1974, synthetic aperture radar interferometry technology developed rapidly in the following decades, especially in 2001 to 2003, when Ferretti and Berardino [9,10,11], respectively, proposed PS-InSAR (persistent scatterer interferometric SAR, PSI) and SBAS-InSAR (Small Baseline Subset Interferometric SAR). Time-series InSAR technology effectively alleviates the effects of spatial–temporal decorrelation and atmospheric delay, significantly increasing the accuracy of InSAR deformation monitoring, making InSAR more widely used in the monitoring of surface deformation [12] and effectively compensating for the labor-intensive and high-cost disadvantages of the traditional surface subsidence monitoring methods [13].

In recent decades, there has been a significant increase in the utilization of satellites for scientific research, leading to advancements in the precision of surface deformation detection through satellite-borne radar [14]. SAR sensors are commonly categorized into three bands: the C-band (e.g., Sentinel-1, FC-1, RADASAT), L-band (ALOS-2, LT-1, etc.), and X-band (COSMO-SkyMed, TerraSAR-X). The X-band excels in accurately capturing subtle deformations in targets but possesses weaker penetration capabilities; on the other hand, the L-band demonstrates stronger penetration capabilities, enabling it to penetrate vegetation canopies and the soil surface. Positioned between the X-band and the L-band, the C-band effectively combines surface information and penetration capabilities [15,16,17]. In order to meet the growing demand for high timeliness in the remote sensing application market, China has begun to establish a large-scale constellation of small and light commercial SAR satellites for network observation, which has become an important trend in satellite remote sensing applications. At the same time, a large number of low-cost and high-quality commercial SAR remote sensing images have entered the market [18,19]. In recent years, the global commercial SAR industry has developed rapidly, with companies such as ICEYE, Capella Space, and Synspective collectively launching 338 Earth observation satellites in 2023. Chinese research institutes such as Tianyi Spacety, Aerospace Macrographic, and China 4D are also rapidly building SAR satellite constellations. Currently, InSAR topographic mapping and InSAR deformation measurement are the main commercial application directions of SAR satellites. With the growing demand for precise surface monitoring in areas such as infrastructure monitoring and natural resource management, the global InSAR market is rapidly expanding.

On 7 June 2023, FC-1 successfully launched and entered the orbit of the Jiuquan Satellite Launch Center in China, with a total weight of only 300 kg. In spotlight mode, the satellite has a resolution better than 1 m, and in strip mode, it has a resolution better than 3 m. Its orbit accuracy is 5–8 cm. In order to utilize FC-1 better in the future, it is urgent to verify the deformation monitoring capability of FC-1.

Many scholars have verified the reliability of PSI technology in monitoring urban deformation: Dumka et al. used the PSI method to monitor the ground subsidence in Ahmedabad, western India, by utilizing Sentinel-1 datasets to obtain ground subsidence data for the study area from 2017 to 2020, with their results showing good consistency with GNSS height data [20]; Duffy et al. used a Sentinel-1 dataset to obtain subsidence information for Ho Chi Minh City, with the distribution of the results for the average subsidence rate basically being consistent with the results of other studies [21]; Kim et al. used the L-band satellite JERS-1 and the PSI technique to obtain surface subsidence data for selected locations in Inchon Port and Busan, with subsidence rates reaching up to 30 mm/year [22]. Guzman et al. used PSI technology and a calibrated finite element model to estimate the safety index of a bridge [23], Macchiarulo et al. combined the PSI technique with a structural assessment to evaluate the damage to buildings caused by subsidence near tunnel excavation sites [24], Guzman et al. developed a decision support system that integrated the PSI technique to improve the quality of road infrastructure [25], Zhang et al. used PSI in combination with an SBAS to study the deformation trends in an airport area over different years [26]. And the accuracy of PSI technology is superior to that of SBAS-InSAR in areas with more urban buildings [27,28,29]. However, although the study area is densely populated with buildings, there are still some areas of sparse vegetation. PSI technology cannot obtain effective results in these areas covered by vegetation. For such areas with moderate coherence, Ferretti [30] and others proposed a DSI algorithm for increasing the density of the measurement points in the DS (distributed scatterer) feature region. DSI technology has also been widely applied in deformation monitoring, and its accuracy has been verified. Li et al. analyzed the surface deformation of Yuxi City using DSI technology and validated the reliability of DSI through comparisons with PSI and SBAS technologies [31]. Tian et al. used DSI technology to obtain subsidence data from non-urban areas. The results of this method were highly consistent with the monitoring results of the StaMPS method. The point density obtained using this method was 17.28 times that of the StaMPS method [32]. Ma et al. applied an improved MT-InSAR technique to extracting PSs and DSs, achieving a high density of deformation points. They then used numerical simulation to model the time-series subsidence of Jingli Building from 2012 to 2016 and 2010 to 2014, which showed good agreement with the InSAR measurements [33]. To comprehensively evaluate the capability of FC-1 in time-series deformation monitoring, this study employs both PSI and DSI algorithms to validate FC-1’s performance in time-series deformation monitoring.

Given that there are currently few research works on the time-series monitoring performance of the FC-1 satellite, this experiment collected and processed data from 20 scenes of the FC-1 satellite (12 October 2023, to 30 May 2024) and 20 scenes of the Sentinel-1 satellite (10 October 2023, to 6 June 2024). The results of the two satellites were compared, and leveling survey data from the study area were used to verify the accuracy of the FC-1 satellite data. The aim was to explore the feasibility of using the FC-1 satellite for temporal InSAR in urban areas and to provide a reference for the future application of the FC-1 satellite in urban subsidence and building facility safety.

## 2. Materials and Methods

### 2.1. The Study Area

**Figure 1:**
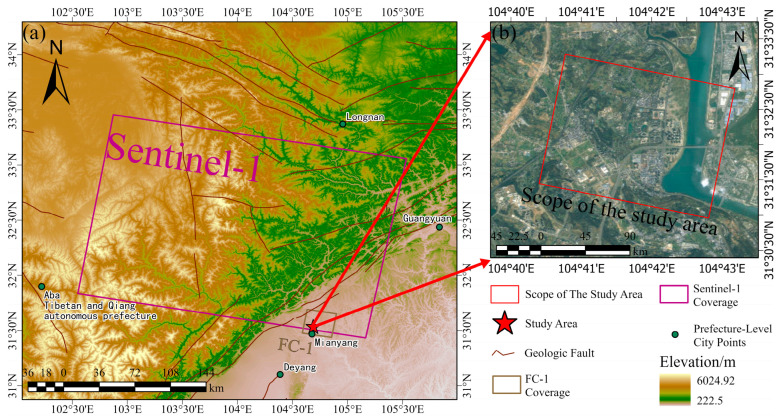
(**a**) Coverage areas of Sentinel-1 (purple) and FC-1 (brown), study area location marked by a five-pointed star, and COPDEM topographic map. (**b**) Google Maps image of the study area.

The research area is located in Mianyang, Sichuan Province, China, the only Science and Technology City in China, also known as Fucheng, after which the FC-1 satellite is named. The region’s altitude varies significantly, with peaks of 2345 m in the northwest and dips down to 501 m in the southeastern direction. The Longmenshan Fault, running in the northeasterly direction, is the primary influence on the landscape. The Songpan–Ganzi Fold System is found to the west, while the Sichuan Basin is situated to the east [34]. The geographical layout of the campus extends 2000 m from east to west and 1500 m from north to south, covering a total area of 2.72 × 10^5^ m^2^. The total building area of the campus is 1.15 × 10^6^ m^2^. The topography of the entire campus is characterized by higher elevation in the west and lower elevation in the east, with the slope of the original ground ranging from 10 to 30°. Both the campus and the surrounding area are used as the study area, and Sentinel-1 covering the study area is used to make a comparison with the FC-1 satellite, which is shown in Figure 1. The campus is located in a hilly area with a gently sloping topography, which is characterized by a shallow slope with a hilly slope geomorphology. Geologic divides are traversed within the area, and the main geologic compositions are grayish-yellow and purplish-red chalky clay, sandy mudstone, fine sandstone, and chalky clay containing sandy mudstone fragments. The study area is characterized by mild climatic conditions and abundant rainfall, with an average annual rainfall of 963.2 mm. The rainfall characteristics include a concentration of rainfall over a short period, heavy rainfall over several consecutive days, and periods of concentrated rainfall.

### 2.2. Datasets

FC-1, the first satellite in the Mianyang Constellation and the first spacecraft manufactured in Sichuan Province, China, was successfully launched on 7 June 2023. The satellite carries a 5.40 GHz C-band SAR payload with left- and right-view capabilities and adopts VV polarization. Its interferometric revisit period is 11 days, and it operates in a sun-synchronous orbit. The FC-1 satellite achieves an orbiting accuracy of better than 10 cm, with an interferometric baseline of re-orbit of less than 300 m, an orbital inclination of 97.4°, and a maximum acquisition length of up to 2000 km.

In strip mode, its spatial resolution reaches 3 m, with its standard scene covering an area of 25 km^2^. In cluster beam mode, its spatial resolution is better than 1 m, and the standard scene covers an area of 7 km^2^. Distance ambiguity has long been a major technical challenge limiting spaceborne SAR in achieving high-resolution wide-swath (HRWS) imaging [35]. FC-1 addresses this issue by optimizing its antenna pattern to reduce the power of the sidelobe signals, thereby alleviating the distance ambiguity caused by sidelobe interference and improving the imaging quality in its SAR images. The successful launch of this satellite marks an important step for China in the field of aerospace, and it can provide high-resolution remote sensing data for many fields such as early warnings of geological disasters [9], environmental monitoring [36], traffic management [12], etc., and provide strong support for the development of related industries. A total of 20 scenes of SLC data from FC-1, provided by Tianyi Spacety, were used in this experiment. PSI and DSI technologies were used to monitor the deformation of the study area. The FC-1 data used in this paper are shown in Table 1.

Sentinel-1 is an Earth observation satellite operated by the ESA, carrying a C-band radar, providing all-weather continuous data acquisition with a 250 km width and a 5 × 20 m resolution, and adopting TOPS technology, which is rich in data and easy to download [37]. In order to ensure that the periods monitored by the two satellites were similar, 20 scenes of data in total from 10 October 2023 to 6 June 2024 were selected for this study, and the specific information is shown in Table 1. The reference DEM used in this study is the Copernicus DEM with a resolution of 30 m [38]. The Copernicus DEM is based on the WorldDEM product and was generated using edited and smoothed radar satellite data obtained from the TanDEM-X mission; the DEM was acquired from 2010 to 2015 [39], and every two years, the ESA makes a minor update to correct it. The DEM data used in this study are the DEM data corrected by the ESA for 2023. In addition, this study collected GACOS data corresponding to image times from the two satellites for atmospheric correction. GACOS is the Generalized Atmospheric Correction Online Service for interferometric synthetic aperture radar (GACOS) product, which provides near-real-time global coverage of the atmospheric time lag and a new method for repeating atmospheric corrections via InSAR [40]. The collection of unmanned aerial vehicle (UAV) oblique photogrammetry data in the study area in July 2023 included UAV oblique three-dimensional models and high-resolution Digital Orthophoto Map (DOM) images. These data contributed to the analysis of the performance of FC-1 [41].

### 2.3. Methods

**Figure 2:**
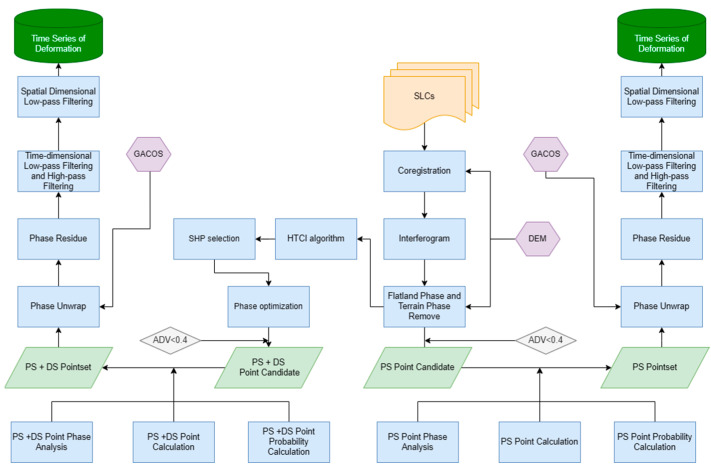
Flow chart of DSI and PSI.

The main commonly used types of time-series InSAR are SBAS-InSAR [11], PSI [10], DS-InSAR [30], and various types of MT-InSAR [42,43,44]. It is more challenging to perform unwrapping in densely built-up areas using high-resolution SAR data, such as TSX, COSMO-SkyMed, and so on. This is because high-rise buildings tend to block the phase continuity, resulting in a decreasing phase from near to far, making the phase of high-rise buildings differ from that of the street level. When a large number of high-rise buildings are present in an area, the deconvolution process may be confused with strong phase abruptness, which is related to the proportion of high-rise buildings in the overall image [45]. This phase mutation is not consistent with reality, and it may be removed during the unwrapping process, resulting in the loss of phase information on the high-rise buildings. On the other hand, the PSI and DSI methods rely on identifying PSs and DSs and performing interferometry and unwrapping for these points [46]. This approach helps to avoid the errors in unwrapping caused by the phase discontinuities that commonly occur when using the SBAS method with high-resolution SAR imagery in urban areas. The FC-1 and Sentinel-1 data processing flow is shown in Figure 2.

**Figure 3:**
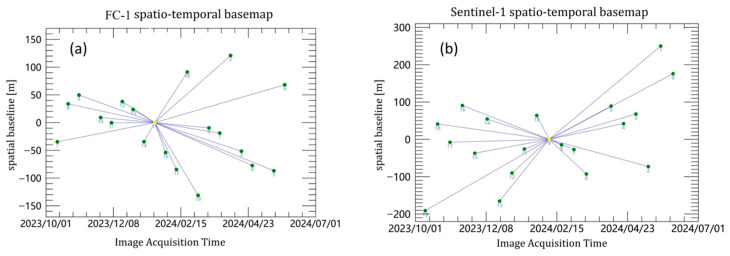
(**a**) Spatio-temporal baseline map of FC-1 single master image. (**b**) Spatio-temporal baseline map of Sentinel-1 single master image.

Firstly, Sentinel-1 images were co-registered to an image acquired on 7 February 2024 by iteratively applying intensity matching and using the “spectral diversity” method, achieving a co-registration accuracy of one-thousandth of a pixel. For FC-1, all of the images were co-registered to an image acquired on 19 January 2024 using a resampling method, with a co-registration accuracy of 0.2 pixels deemed sufficient to meet the interferometric processing requirements. To ensure consistency, images from both satellites covering similar time periods were selected for the time-series analysis, and master images were chosen from similar time periods during the processing. For the FC-1 satellite and Sentinel-1, we used images from 19 January 2024 and 7 February 2024 as the respective master images, with the resulting spatio-temporal baseline maps presented in Figure 3.

Following the selection of interferometric pairs, differential interferometry was applied to the images. The interferograms were subsequently processed to eliminate the effects of flatness and topography by utilizing an external Digital Elevation Model (DEM). After obtaining flattened interferograms following the PSI method, we selected PS points based on the deviation in amplitude and estimated phase and then performed time-series processing to derive the deformation time series. In DSI, after flattened interferograms are obtained, phase optimization is applied to enhance the phase quality of the DS points to the level of PS points, enabling the same processing steps to be followed for DSI as PSI in subsequent analyses.

In this study, the FC-1 and Sentinel-1 satellites were employed, using PSI and DSI methods to obtain the time-series subsidence in the study area from October 2023 to June 2024. A cross-validation comparison of the two methods and satellites was conducted to verify the capability of the new-generation small SAR satellite, FC-1, in time-series deformation monitoring, and the performance of the DSI and PSI methods was compared. This analysis aims to provide a reference for the future application of FC-1 in urban deformation monitoring. The following sections, Section 2.3.1 and Section 2.3.2, outline the key procedures of PSI and DSI in this article.

#### 2.3.1. Processes and Principles of PSI

PSI was created by Ferretti et al. in 2001. It works by looking at the phase information in a set of SAR images to find points called permanent scatterers that keep the time series coherent. In 2007, Hooper et al. proposed a method named StaMPS [46], which is a type of PSI. The StaMPS-PS method combines the amplitude deviation threshold and the phase stability to select stable PS points. The “traditional” PSI typically requires at least 25 interferograms to obtain reliable results, while StaMPS only needs 12, making it more efficient. Therefore, this study adopts the StaMPS method for PS processing. Firstly, PS candidate points are selected using the Amplitude Dispersion Index ($D_{A}$), defined as follows:

(1) $$D_{A}=\frac{\sigma_{A}}{\mu_{A}}$$

where $\sigma_{A}$ and $\mu_{A}$ are the standard deviation and average of a series of amplitude values, respectively. In this study, the ADV (the threshold of the Amplitude Dispersion Index) was set to 0.4. This low threshold ensured that almost all potential PS pixels are included. However, it also resulted in the selection of many non-PS pixels. Therefore, an additional analysis of the phase stability and PS probability calculations were performed to eliminate these non-PS pixels effectively.

Due to the 3 m resolution of FC-1, different parameters are required in the StaMPS 4.1 software for candidate point selection compared to those for Sentinel-1. For initial PS point selection, both satellites utilize an ADV of 0.4. However, during the subsequent phase noise estimation and PS selection process, distinct parameters must be set for each satellite. We employed the “percent” method for PS point selection, setting the acceptable spatial percentage of selected pixels per square kilometer to 1% for FC-1, while for Sentinel-1, this parameter was set at 20%. It was observed that for high-resolution satellites like FC-1, applying the default setting of 20% results in no PS points being selected, which is clearly incorrect.

In the subsequent “PS_weed” step, pixels with excessive noise or contributions from adjacent ground resolution elements were discarded. Here, the “weed_standard_dev” parameter was set to 1.2 for Sentinel-1, while a more stringent value of 0.8 was used for FC-1. Due to FC-1’s high resolution, a larger number of PS points are selected, allowing many points to remain even when the stricter threshold is applied. This step only discards a few points for urban buildings but removes a large number of noisy and inaccurate points, thereby reducing the computational load in urban environments and improving the quality of the PS points. If Sentinel-1 were set to 0.8, many PS points on buildings would be removed, hindering further analysis. The key parameters of the StaMPS process are listed in Table 2.

**Figure 4:**
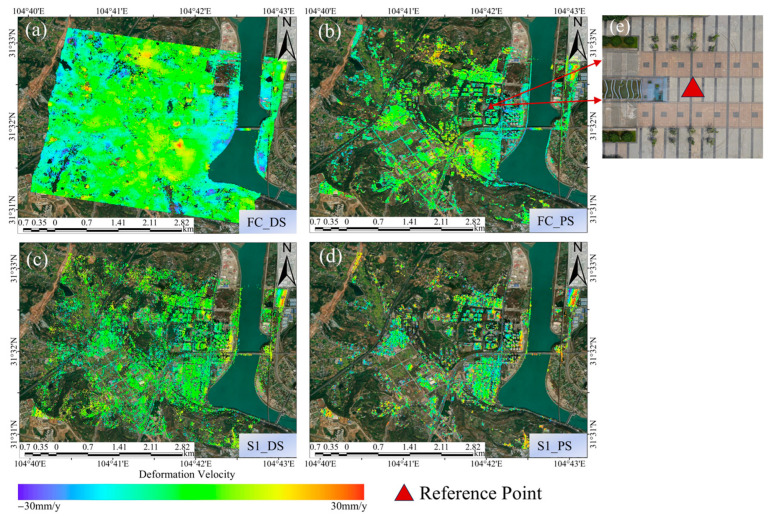
(**a**,**b**) Vertical deformation velocity maps from FC-1 using the DSI and PSI methods. (**c**,**d**) Vertical deformation velocity maps from Sentinel-1 using the DSI and PSI methods. (**e**) Drone orthophoto of the reference point.

After PS point selection, 3D phase unwrapping is performed, followed by the removal of the atmospheric phase delay using GACOS. The selection of the reference points is critical to ensure the accuracy of the time-series deformation measurements. Since InSAR measures the relative displacement, choosing a reference point within a subsiding area can lead to a perceived uplift in stable regions, significantly compromising the accuracy of the results [47]. Ideally, the reference points should be located in areas with minimal topographic variation, preferably on flat surfaces, and should exhibit low phase and deformation gradients, as well as a stable imaging performance. Based on these considerations, a stable artificial platform within the study area was selected as the reference point (see Figure 4). Finally, following time-series processing, the final deformation results were obtained.

#### 2.3.2. Processes and Principles of DSI

The critical stages of DSI include SHP (Statistically Homogeneous Pixel) selection and phase optimization. Distributed targets are usually represented by neighboring pixels with uniform scattering characteristics that follow the same statistical distribution in time-series SAR images. For the SHP selection, we used the HTCI method proposed by Jiang et al. [43,44], a parameter-based testing approach that significantly enhances the testing speed compared to traditional non-parameter testing methods, such as the Kolmogorov–Smirnov (KS) test. Given the differing resolutions of the two satellites, selecting the SHPs required setting varying window sizes to achieve the optimal phase optimization results. The window size should be inversely proportional to the azimuth and range resolutions [43,44], leading us to set the window size for FC-1 to 55 × 55 and that for Sentinel-1 to 11 × 55. As for the phase optimization, we adopted the EMI (efficient phase estimation for interferogram stacks) method proposed by Ansari et al. [48], which incorporates the weights of maximum likelihood (ML) estimators to improve the phase estimation accuracy. The feature decomposition *C* and *T* can be represented as follows:

(2) $$C=\sum_{i=1}^{N}\lambda_{i}v_{i}v_{i}^{H}$$

(3) $$T=\sum_{i=1}^{N}\lambda_{i}\mu_{i}\mu_{i}^{H}$$

where C is an *N* × *N*-dimensional covariance matrix, used to represent the scattering information on the pixels; T is an *N* × *N*-dimensional coherence matrix used to represent the coherence information on the pixels; $\lambda_{i}$ is the eigenvalue; and $\lambda_{1}>\lambda_{2}>\cdots >\lambda_{N}$; $v_{i}$ and $\mu_{i}$ are the eigenvalues to $\lambda_{i}\;$as the optimized phase, while the other eigenvectors can be regarded as noise, and then

(4) $$\theta_{M-C}=argmax\left\{v_{1}^{H}Cv_{1}\right\}$$

(5) $$\theta_{M-C}=argmax\left\{v_{1}^{H}Cv_{1}\right\}$$

where $\theta_{M-C}$ is the phase value optimized on the covariance matrix C through matrix decomposition; $v_{1}$ is the eigenvector of the maximum eigenvalue corresponding to C; $\theta_{M-T}$ is the phase value optimized on the covariance matrix T through matrix decomposition; and $\mu_{1}$ is the eigenvector of the maximum eigenvalue of T.

Phase optimization not only preserves the original resolution of SAR images but also suppresses phase noise, resulting in the achievement of a comparable quality in the DS candidate points to that of the PS points. Further point selection, 3D unwrapping, and time-series processing were then conducted using the StaMPS package, resulting in time-series data for both the DSs and PSs. The parameter settings and processes for DSI time-series processing were consistent with those used in PSI (the key parameters are set in Table 2).

## 3. Results and Analysis

### 3.1. Comparison of the Surface Deformation Distribution

The results of InSAR deformation are generally verified using GNSSs, leveling instruments, etc. In addition to the verification of the PS points detected by the FC-1 satellite using leveling, Sentinel-1 data were also used for comparative verification in this experiment to compare the effect of two kinds of satellite deformation monitoring. The deformation velocity obtained by PSI processing using the FC-1 satellite data and the Sentinel-1 satellite data is LOS (radar line-of-sight) deformation, and in order to facilitate the analysis and comparison of the results of the two types of satellites, the LOS deformation was uniformly projected as vertical deformation. We assumed that no horizontal deformation existed in the region and that radar line-of-sight displacements were caused entirely by the vertical deformation of the ground surface [49]. The specific transformation formula is as follows:

(6) $$d_{V}=\frac{d_{LOS}}{cos\left(\theta \right)}$$

where $d_{V}\;$is the vertical deformation velocity, $d_{LOS}$ is the LOS deformation velocity, and θ is the satellite’s angle of incidence.

The results of converting the deformation velocity of the two satellites into the vertical direction are shown in Figure 4. Through analyzing the deformation velocity map, it can be found that the high resolution of FC-1 results in a markedly higher density of PS and DS points during PSI and DSI processing compared to the density obtained by processing the Sentinel-1 satellite data. Specifically, the FC-1 satellite collected 241,696 PS points and 577,047 DS points, while Sentinel-1 obtained only 18,076 PS points and 32,263 DS points. The former yielded 13.4 times more PS points and 17.9 times more DS points than the latter, and more points facilitate a clearer observation of the deformation distribution.

**Figure 5:**
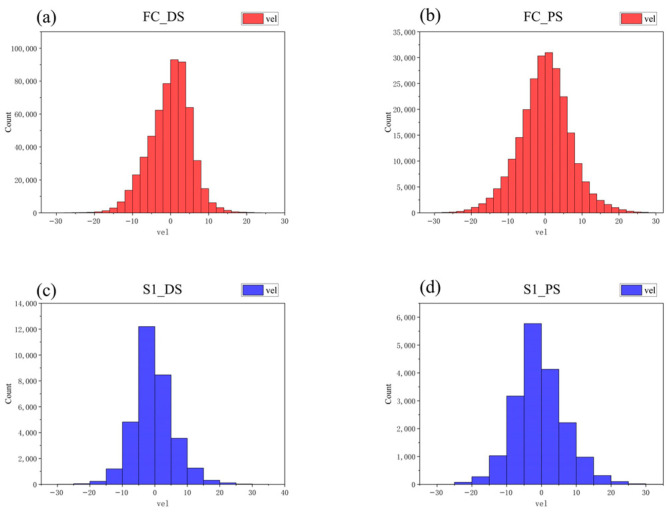
(**a**,**b**) Histograms of deformation velocity from FC-1 using the DSI and PSI methods. (**c**,**d**) Histograms of deformation velocity from Sentinel-1 using the DSI and PSI methods.

To demonstrate the deformation conditions of the area in a more intuitive manner, we calculated histogram statistics of the deformation velocity of the two satellites. As shown in Figure 5, the number of PS points and DS points near 0 mm/y is the highest for both satellites, and they are both normally distributed. The average deformation velocity (with the results rounded to two decimal places) of FC-1’s DSI results is −0.13 mm/y, and the average deformation velocity of FC-1’s PSI results is −0.06 mm/y. For Sentinel-1, the average deformation velocity of its DSI results is −0.13 mm/y, and the average deformation velocity of Sentinel-1’s PSI results is −0.43 mm/y. The statistical results indicate that the deformation velocity in the study area is generally between −30 mm/y and 30 mm/y (positive values indicate uplift, while negative values indicate subsidence). As shown in Figure 4, the monitoring results of the two satellites indicate that the study area was stable during the monitoring period overall, without significant widespread deformation.

**Figure 6:**
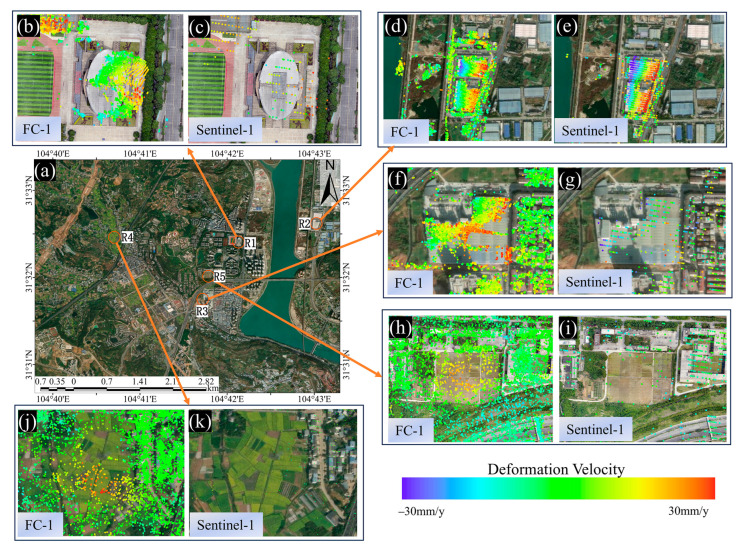
(**a**) Schematic diagram of the research area on Google Earth. (**b**,**c**) Deformation rate maps of region R1 obtained by FC-1 and Sentinel-1 using the PSI method, with a drone image as the base map. (**d**–**g**) Deformation rate maps of regions R2 and R3 obtained by FC-1 and Sentinel-1 using the PSI method, with Google Earth as the base map. (**h**–**k**) Deformation rate maps of regions R4 and R5 obtained by FC-1 and Sentinel-1 using the DSI method, with Google Earth or a drone image as the base map.

However, in terms of the distribution of surface deformation (as shown in Figure 6), both satellites detected more severe deformations in areas R1 and R2. Area R1 is the gymnasium within the school, which was completed in 2010 and is located next to a main road with heavy truck traffic. It is also adjacent to a construction site, posing deformation risks. Area R2 is a logistics park where significant westward subsidence and eastward uplift have been observed. In addition, FC-1’s DSI results also show larger deformations in areas R3, R4, and R5. Area R3 is a building material factory, where Sentinel-1 also detected an uplift trend, although this was not as evident as in FC-1’s results. Areas R4 and R5, as observed from satellite images and 3D models, are farmlands demonstrating noticeable deformations, which is normal. These two locations are typical distributed scatterers, illustrating the superior performance of DSI technology, as it can effectively detect areas of low vegetation and soft soil in addition to buildings. These results preliminarily validate the consistency of the FC-1 and Sentinel-1 satellites’ results.

### 3.2. Point Coverage Comparison of the PSI Method

**Figure 7:**
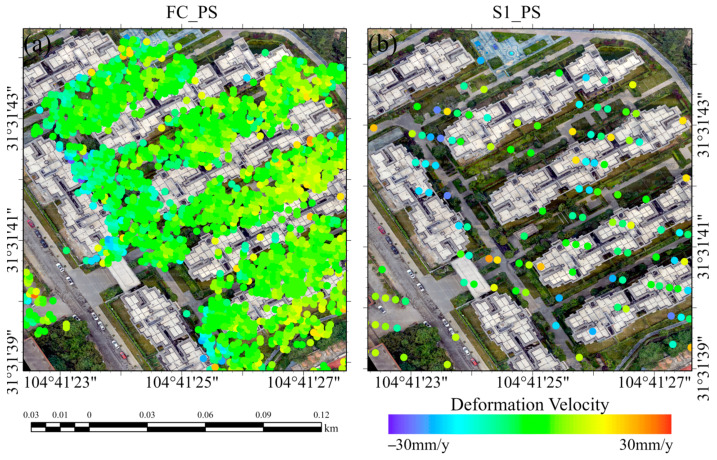
(**a**) Deformation velocity points obtained by FC-1 using the PSI method overlaid onto a drone image. (**b**) Deformation velocity points obtained by Sentinel-1 using the PSI method overlaid onto a drone image.

**Figure 8:**
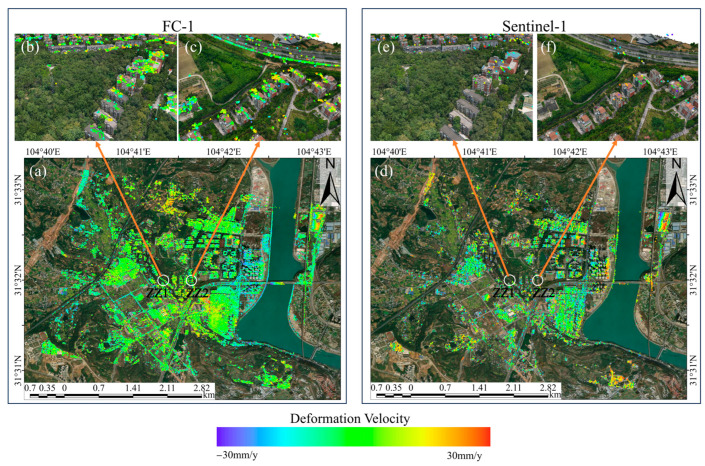
(**a**,**d**) Deformation velocity maps from FC-1 and Sentinel-1 using the PSI method, with schematic maps of ZZ1 and ZZ2 locations. (**b**,**c**) PS deformation points from FC-1 overlaid onto drone oblique images of ZZ1 and ZZ2. (**e**,**f**) PS deformation points from Sentinel-1 overlaid onto drone oblique images of ZZ1 and ZZ2.

In the overall analysis of the distribution of the deformations, the processing results of the FC-1 satellite demonstrate greater continuity. Upon detailed examination, the distribution of PS points on artificial buildings is superior for the FC-1 satellite compared to that of Sentinel-1. Furthermore, the processing results of FC-1 reveal a higher density of PS points covering the entire structure of the buildings, whereas the density of PS points in the results of Sentinel-1 is lower (as shown in Figure 7), with some buildings not being covered at all. Additionally, the coverage of FC-1 in the same area exceeds that of Sentinel-1. Combining high-resolution optical remote sensing images and UAV image analysis, Sentinel-1 cannot detect certain permanent scatterers in the area of artificial buildings, as shown in Figure 8. Sentinel-1 fails to obtain effective results in the ZZ1 and ZZ2 areas, while the FC-1 satellite is still able to calculate PS points on these buildings. This suggests that in urban deformation detection, the FC-1 satellite with a 3 m high resolution can identify building deformations more effectively. Not only is it capable of detecting more buildings in the same area but it can also specifically determine which part of a building has undergone deformation. These advantages in terms of higher point density and coverage will be further analyzed and discussed in Section 3.3.

### 3.3. Point Coverage Comparison of the DSI Method

The DSI method used in this study combined PSs and DSs, with the point densities using DSI with FC-1 and Sentinel-1 being 2.4 times and 1.8 times those when using PSI, respectively. As shown in Figure 4, after phase optimization, the area of the coverage of the detection points from both satellites significantly increased, especially for FC-1, where severely deformed locations can clearly be observed. This section focuses on analyzing and comparing the point coverage under the DSI method for both satellites, as well as the advantages of high-density and high-coverage PSs and DSs.

**Figure 9:**
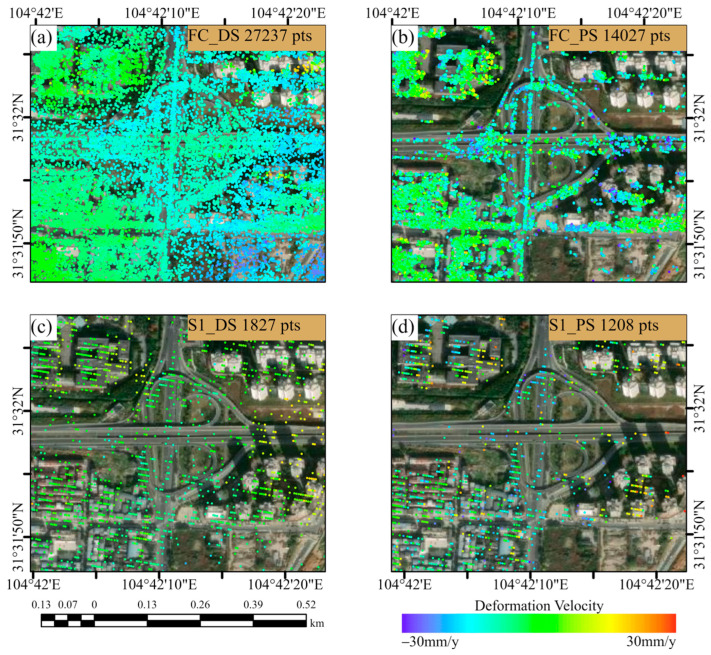
(**a**,**b**) Deformation velocity points obtained by FC-1 using DSI and PSI methods overlaid onto Google imagery. (**c**,**d**) Deformation velocity points obtained by Sentinel-1 using DSI and PSI methods overlaid onto Google imagery.

**Figure 10:**
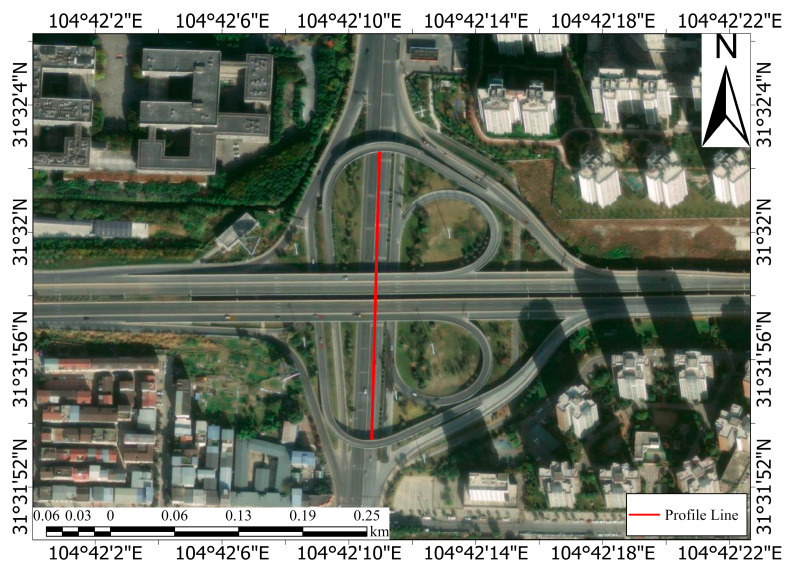
Diagram of road profile location.

**Figure 11:**
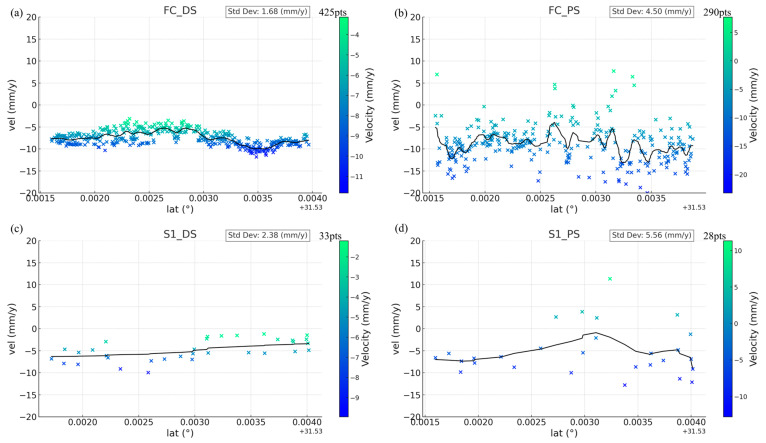
(**a**,**b**) Deformation velocity profile of FC-1 under the DSI and PSI methods. (**c**,**d**) Deformation velocity profile of Sentinel-1 under the DSI and PSI methods.

A notable feature of FC-1 is its higher resolution compared to that of Sentinel-1, which enables more accurate capture of deformation details, especially when using the DSI method (as shown in Figure 9). The dense PS and DS points obtained from FC-1 using the DSI method provide comprehensive coverage of the entire interchange bridge, allowing for effective detection of local deformations. Using both the PSI and DSI methods, we obtained deformation velocity profiles for the same area from both FC-1 and Sentinel-1. The selection criterion involved considering only points within 10 m of the profile line (as shown in Figure 10). These profiles demonstrate that after phase optimization, the DSI method increases the number and density of the monitoring points, enhancing the continuity of the profile and reducing the dispersion of the deformation velocity points. As shown in Figure 11, the DSI method significantly reduces the dispersion of the deformation velocity profiles for both satellites, with values decreasing from 4.50 mm and 5.56 mm to 1.68 mm and 2.38 mm, respectively. This reduction in dispersion makes it easier to confirm that the deformation velocity for this section of the road ranges from −10 mm/y to 0 mm/y. Additionally, the higher point density improves the profile continuity. As illustrated in Figure 9, the dense PS and DS points obtained using FC-1 via the DSI method enable accurate measurement of the deformation velocity at specific locations on the road, eliminating the need for interpolation to estimate these values. The combination of FC-1’s high-resolution data and the DSI method provides a powerful tool for monitoring urban surface deformations.

**Figure 12:**
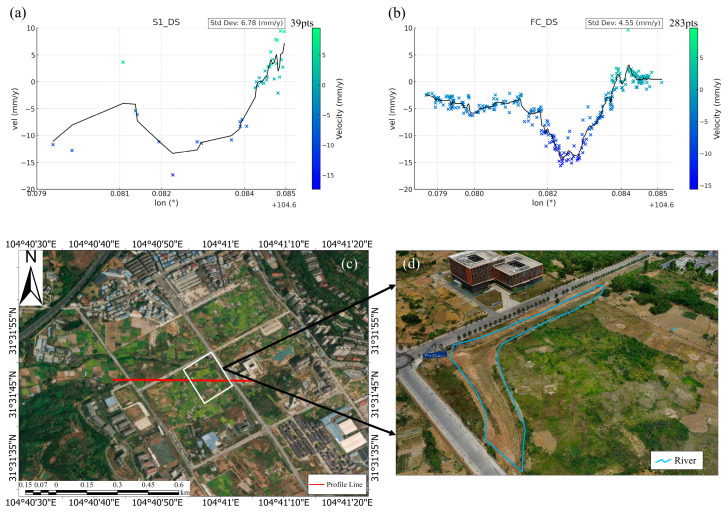
(**a**,**b**) Deformation rate profiles of Sentinel-1 and FC-1 under the DSI method. (**c**) Diagram of position of vegetation section line. (**d**) UAV 3D model of vegetation area.

The DSI method has been proven effective for monitoring deformation in sparsely vegetated areas, where the PSI method fails to produce results [30]. In regions with sparse vegetation, FC-1 also demonstrates excellent detection performance. As depicted in Figure 12d, a vegetated area is located near a small river, where the soft soil poses a significant risk of subsidence. To analyze this further, we selected points within a 10 m radius of the profile line shown in Figure 12c and generated deformation velocity profiles for both FC-1 and Sentinel-1, as illustrated in Figure 12a,b. In Figure 12b, the FC-1 results clearly reveal a well-defined funnel of rapid subsidence, while Sentinel-1 shows a similar trend but with fewer data points near the funnel’s center (as shown in Figure 12a). This scarcity of points limits Sentinel-1’s ability to provide comprehensive monitoring in sparsely vegetated areas, making it less effective than FC-1 in accurately capturing the deformation pattern in such regions. Additionally, while the DSI method enables the detection of deformation in vegetated areas, the deformation velocity points in these regions appear more dispersed compared to those in non-vegetated areas. Despite this limitation, having data, even if it is more dispersed, is still significantly better than having no data at all.

### 3.4. Accuracy Evaluation

InSAR detection results are currently verified for their reliability by using ground test data such as leveling measurements or GPS. In this section, we evaluate the accuracy of the capability of FC-1 in temporal deformation monitoring using both internal consistency assessment and external consistency assessment methods, starting from the data processing step and comparing the different monitoring technologies.

#### 3.4.1. Evaluation of the Internal Coincidence Accuracy 

**Figure 13:**
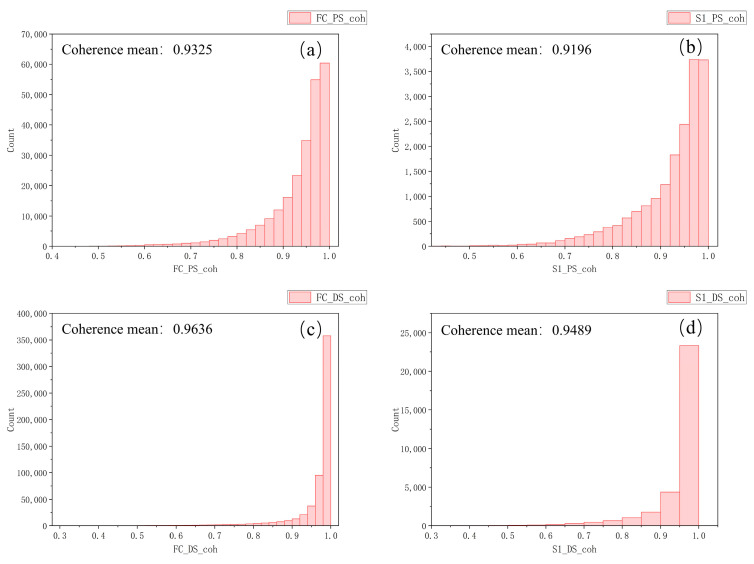
(**a**,**b**) Coherence histograms and average coherence values for the PSI method with FC-1 and Sentinel-1. (**c**,**d**) Coherence histograms and average coherence values for the DSI method with FC-1 and Sentinel-1.

We evaluated the accuracy internally based on two standards: the standard deviation of coherence and velocity estimates (relative to the average strain rate). Coherence reflects the similarity between signals and is an important indicator for assessing the quality of InSAR data. The coherence value ranges from 0 to 1, with higher values indicating more reliable data and greater precision in the deformation measurements. Various factors, such as the time interval between images, satellite geometry, surface features, and atmospheric effects, influence image coherence. The average coherence of the points obtained from FC-1 using the DSI and PSI methods is 0.964 and 0.932, respectively (as shown in Figure 13). In comparison, the average coherence for Sentinel-1 is 0.949 and 0.920, respectively. For the DSI method, 91% of the total number of points extracted by FC-1 have a coherence above 0.9, while in the case of the PSI method, this is 78%. Regarding using Sentinel-1 with the DSI method, the percentage of points with a coherence above 0.9 is 86%, and for the PSI method, this is 72%.

**Figure 14:**
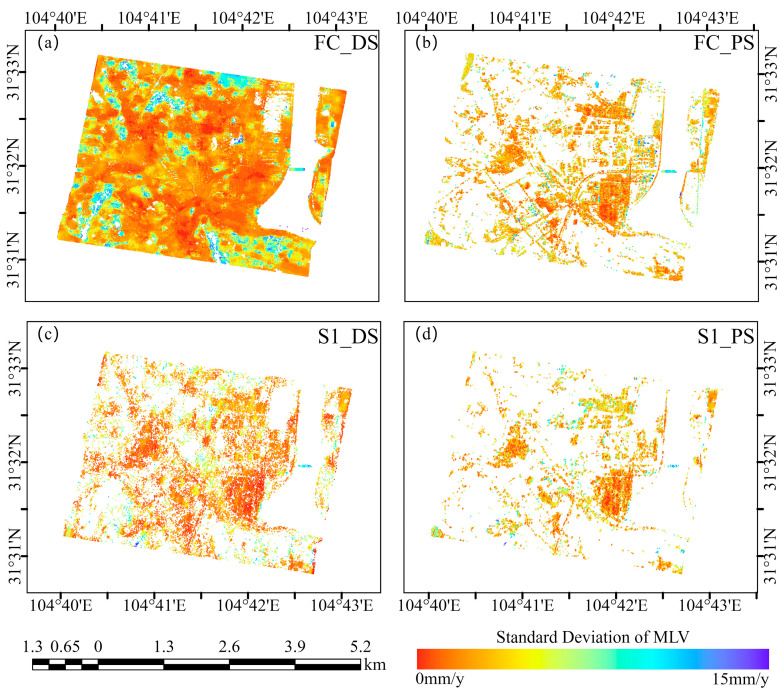
(**a**,**b**) Standard deviation maps of deformation velocity for Sentinel-1 using PSI and DSI methods. (**c**,**d**) Standard deviation maps of deformation velocity for FC-1 using PSI and DSI methods.

The StaMPS software package outputs the standard deviation in the mean LOS velocity (MLV). A higher standard deviation indicates a greater likelihood that a point represents noise, while a lower standard deviation signifies its higher reliability. In Figure 14., we present images showing the standard deviation of the points for FC-1 and Sentinel-1 when using the DSI and PSI methods. Within the study area, FC-1 and Sentinel-1 achieve excellent results under both methods. For both satellites and methods, more than 99% of the points have a standard deviation in the MLV below 1 cm/year. Specifically, using the DSI method, 75% of the points from FC-1 have a standard deviation in the MLV below 5 mm/year, compared to 54% when using the PSI method. In contrast, Sentinel-1 has values of 37% and 54% for the respective methods. The superiority of FC-1 is significant. We calculated the standard deviation in the MLV for FC-1, and the results were 2.40 mm/y using the DSI method and 2.76 mm/y using the PSI method. In contrast, for Sentinel-1, the standard deviation in the MLV is 2.86 mm/y for the DSI method and 3.47 mm/y for the PSI method. Notably, the standard deviation in the MLV for FC-1 is superior to that of Sentinel-1 under both methods.

In conclusion, after undergoing two types of internal accuracy evaluations, both satellites have demonstrated favorable results. Nonetheless, it is apparent that FC-1 outperforms Sentinel-1 based on both evaluation criteria. Notably, the DSI method has substantially enhanced the signal-to-noise ratio of the interferogram. Consequently, the DSI method has not only augmented the point density and coverage but has also enhanced the accuracy of deformation monitoring when compared to that of PSI.

#### 3.4.2. Evaluation of the External Coincidence Accuracy 

External accuracy assessments of InSAR results typically involve the validation of data using ground monitoring techniques such as leveling measurements and GPS. In this study, in addition to using leveling measurements to verify the monitoring accuracy of FC-1, we also selected four regions with significant deformation to examine the correlation between the temporal subsidence measurements of FC-1 and Sentinel-1, aiming to validate the reliability of FC-1’s temporal monitoring.

**Figure 15:**
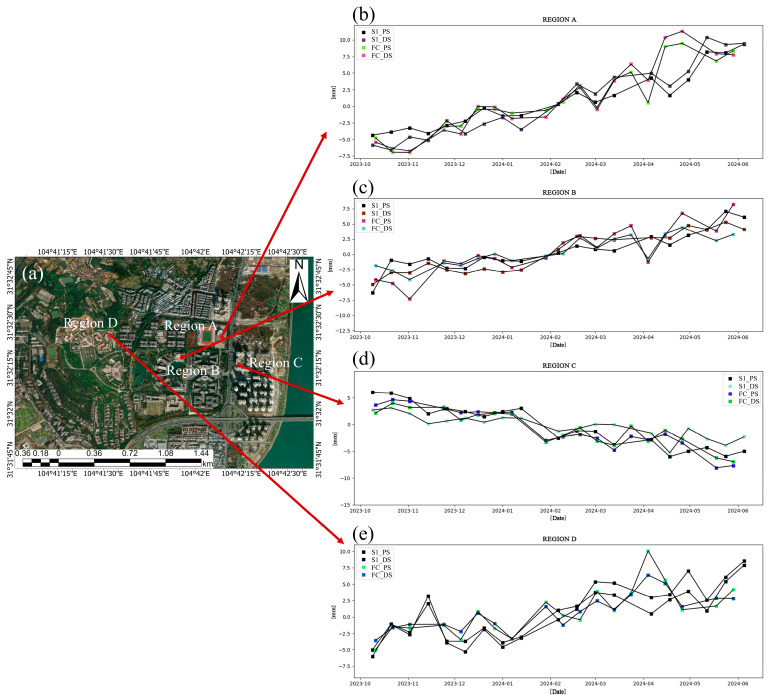
(**a**) Diagram of locations of four regions A, B, C and D. (**b**–**e**) Time-series settlement maps of FC-1 and Sentinel-1 using the DSI and PSI methods in four regions.

**Figure 16:**
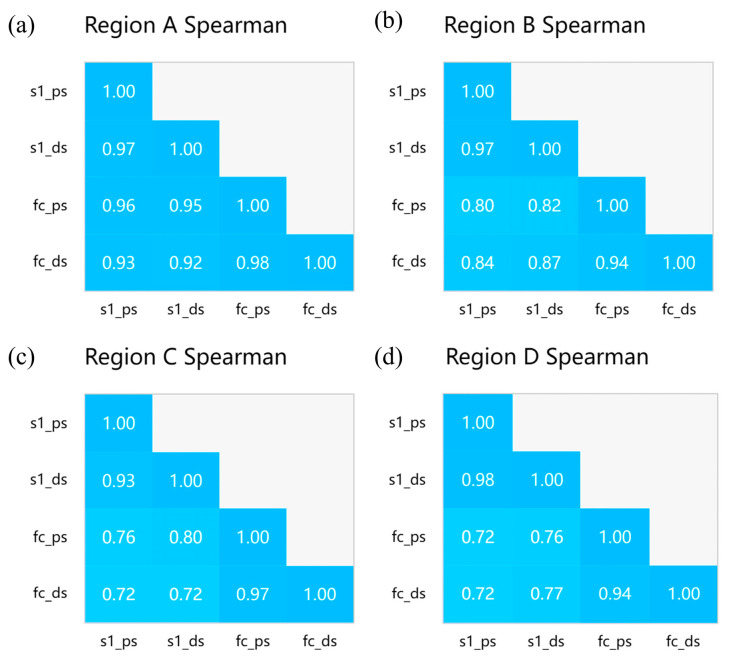
(**a**–**d**) Spearman’s correlation matrix heatmaps of the time-series settlement amounts obtained by FC-1 and Sentinel-1 using the DSI and PSI methods in four regions.

**Figure 17:**
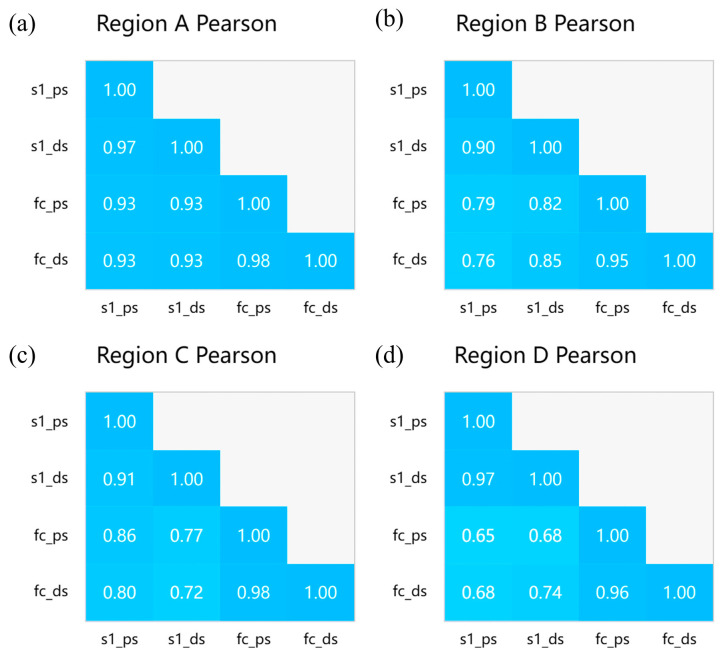
(**a**–**d**) Pearson’s correlation matrix plots of the time-series subsidence values between FC-1 and Sentinel-1 using the DSI and PSI methods in four regions.

As shown in Figure 15, the deformation trends detected by the two satellites in these four regions exhibit similarities. To present the correlation in the results on temporal subsidence more clearly, we conducted an analysis using Spearman’s and Pearson’s methods [50,51] to examine the correlation between the monitoring results of the two satellites. The visualized correlation is presented in Figure 14. Notably, the results from both satellites exhibit a significant correlation, regardless of whether the DSI or PSI method is used. Under the PSI method, the Spearman’s correlation ranges from 0.72 to 0.96, and the Pearson’s correlation ranges from 0.68 to 0.93. As for the DSI method, the Spearman’s correlation ranges from 0.77 to 0.92, and the Pearson’s correlation ranges from 0.74 to 0.93 (as shown in Figure 16 and Figure 17). Additionally, the correlation of the temporal subsidence obtained from the processed DSI and PSI results, post-phase optimization, exceeds 0.9, indicating that phase optimization does not compromise the accuracy of the PS points.

We also compiled the root mean square error (RMSE), maximum differences, minimum differences, and average differences of the temporal settlement obtained by the two satellites using the PSI and DSI methods in Table 3. The results indicate that in the four regions analyzed, the maximum error remains within 1 cm, with a maximum differences of 7.145 mm, and the average RMSE ranges from 0.832 mm to 1.042 mm. The differences between them are all within 1 cm, which demonstrates the consistency of the monitoring results from both satellites.

**Figure 18:**
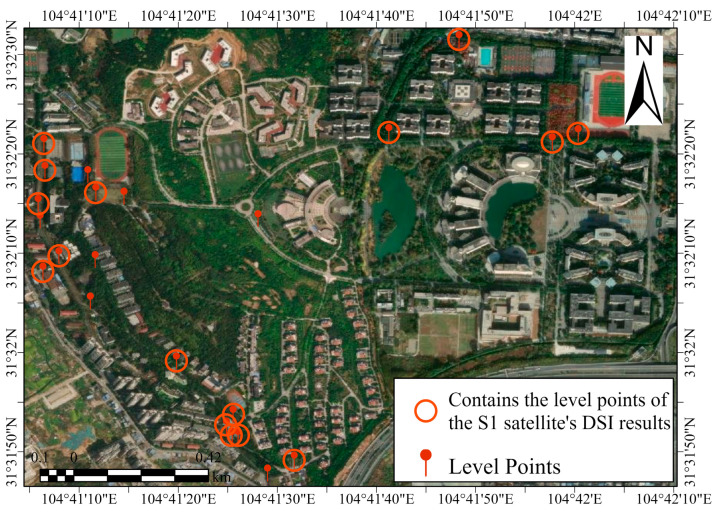
Illustrative Google Earth map showing the locations of level points.

**Figure 19:**
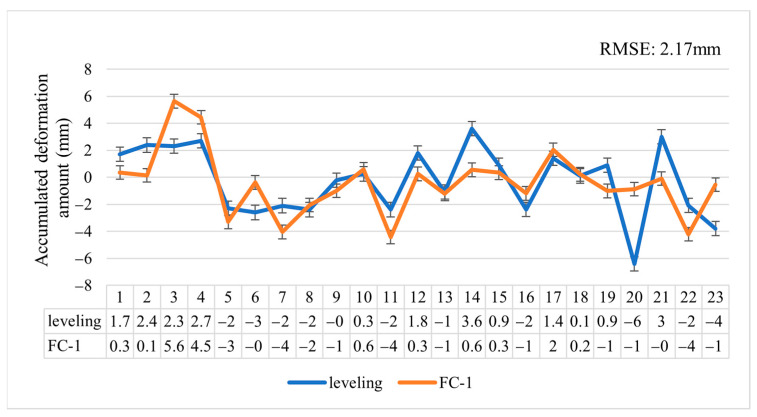
The subsidence measured by FC-1 using the DSI method compared to the subsidence measured by leveling.

For the leveling measurements, we used a Topcon DL501 electronic level, which has an accuracy of 0.3 mm, meeting the requirements for validating InSAR subsidence monitoring. We conducted a round-trip leveling survey in accordance with the second-class leveling standards; obtained data for two periods, 1 October 2023 and 1 June 2024; and calculated the deformation between these two dates. The distribution of the leveling point positions can be seen in Figure 18. Since PSI only yielded a smaller number of PS points, not all of the level points were supported by the PS points. However, in this particular case, the DSI image proved adequate for analysis. We compared the average cumulative deformation of the points within a 10 m radius around the leveling points with the settlement data derived from the leveling measurements. Figure 19 specifically presents the results on vertical displacement obtained from DSI and leveling. As depicted in Figure 19, the settlement measured by FC-1 closely resembles the results from the leveling measurements, with a maximum error of 5.52 mm, an average error of 1.76 mm, an RMSE of 2.17 mm, and a minimum error of only 0.34 mm. This demonstrates that the capability of FC-1 in deformation monitoring achieves a millimeter-level precision, making it suitable for future surface deformation monitoring.

**Figure 20:**
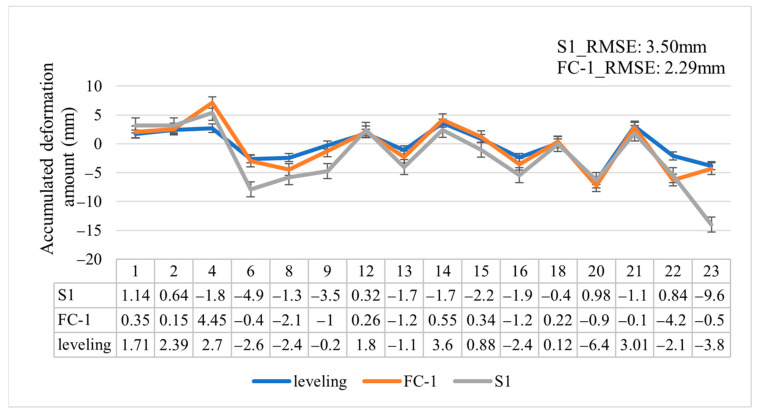
The subsidence measured by FC-1 and Sentinel-1 using the DSI method compared to the subsidence measured by leveling.

In the following analysis, we focus exclusively on the 16 level points for which valid results were obtained by the Sentinel-1 satellite, as 7 other level points remained invalid despite the application of the DSI method (as shown in Figure 18). Additionally, we present the cumulative deformation data for FC-1 and Sentinel-1 and the leveling measurements in Figure 20, enabling a comparison of the deformation accuracy of both satellites. The measurement errors for FC-1 and Sentinel-1 are summarized in Table 4. Sentinel-1 achieved an RMSE of 3.5 mm, a maximum error of 7.38 mm, an average error of 2.83 mm, and a minimum error of 0.32 mm. In comparison, FC-1 recorded an RMSE of 2.29 mm, a maximum error of 5.52 mm, an average error of 1.83 mm, and a minimum error of 0.34 mm. Both satellites demonstrated millimeter-level accuracy in deformation monitoring; however, FC-1 outperformed Sentinel-1 in three key metrics—RMSE, maximum error, and average error. This superior performance is largely attributed to FC-1’s higher density of PSs and DSs, which provide a greater number of monitoring points around each level point location. The increased number of deformation point samples helps to reduce potential errors, while Sentinel-1’s performance is constrained by its lower resolution. Despite the advanced DSI method, Sentinel-1 could not produce valid results for seven level points, whereas FC-1 provided valid results for all points. Furthermore, although Sentinel-1 produced valid results for the 16 remaining points, its RMSE, maximum error, and average error were inferior to those of FC-1. Our analysis concludes that FC-1’s capabilities in deformation monitoring achieve millimeter-level accuracy, demonstrating its strong potential for future applications in both surface deformation monitoring and infrastructure health assessments.

## 4. Discussion

In this study, we evaluated the deformation monitoring capabilities of the FC-1 satellite using PSI and DSI time-series InSAR techniques. The research results reveal several insights into the performance and accuracy of this satellite in detecting surface deformation in urban environments.

First, the FC-1 satellite demonstrates a significant advantage in terms of point density over Sentinel-1, primarily due to its higher spatial resolution. Compared to Sentinel-1, FC-1 exhibits a 12.4-fold increase in the number of PS points and a 16.9-fold increase in the number of DS points, offering more detailed deformation measurements and enabling a finer observation of the deformation patterns in urban areas. This is crucial for infrastructure monitoring, as the higher density of PS and DS points facilitates better detection of deformations in buildings and other man-made structures.

Moreover, the application of both PSI and DSI techniques shows that FC-1 performs better in areas with sparse vegetation and urban environments. Sentinel-1, while effective, is limited in its ability to detect subtle deformations due to its lower resolution. Importantly, Sentinel-1 is unable to acquire PS points on certain buildings, which means that deformation monitoring of these structures is not feasible. This makes FC-1 a more suitable choice for urban planning and disaster management applications, particularly when early detection of small-scale deformations is necessary. In areas characterized by sparse vegetation, FC-1 maintains a high density of deformation monitoring points, whereas Sentinel-1 is ineffective in these regions. This indicates that FC-1 possesses a stronger capability for early warnings of geological hazards in mountainous areas. However, further testing and analysis are needed to fully evaluate FC-1’s deformation monitoring capabilities in mountainous regions.

The accuracy evaluation, based on both internal and external consistency assessments, demonstrates FC-1’s excellent time-series deformation monitoring capabilities, outperforming Sentinel-1 in several aspects. FC-1 exhibits better coherence and a lower standard deviation in its deformation measurements. Comparison with leveling data confirms that FC-1 achieves a millimeter-level precision in deformation monitoring. These findings indicate that FC-1 excels not only in point density but also in data accuracy, making it a powerful tool for surface deformation monitoring.

High-resolution satellites like FC-1 encounter certain limitations when applying SBAS-InSAR technology in densely built areas, as high-rise buildings disrupt the phase continuity. This disruption leads to incorrect unwrapping results and consequently unreliable time-series deformation sequences. In contrast, these issues are not observed in suburban areas.

In summary, the FC-1 satellite has the capability to monitor the surface deformation in urban areas through the application of advanced time-phase InSAR technology. Its high-resolution data combined with the DSI method provide a more comprehensive and accurate assessment of deformation patterns, making it a valuable asset in the field of urban surface monitoring. However, compared to PSI technology, DSI technology requires more time due to the estimation of the full information volume for all possible interferometric pairs. In the future, further improvements to the algorithm and assessments of FC-1’s monitoring capabilities in mountainous and canyon areas are necessary to expand its application in identifying geological disasters in mountainous regions.

## 5. Conclusions

This paper presents an analysis and comparison of the deformation monitoring results from the FC-1 and Sentinel-1 satellites using PSI and DSI techniques. The FC-1 satellite, with its higher resolution (3 × 3 m), yields a denser distribution of PS and DS points than Sentinel-1 (5 × 20 m). However, its higher resolution also means that using SBAS-InSAR in densely built areas may lead to phase continuity disruptions due to high-rise buildings, resulting in unwrapping errors—a problem not encountered by the medium-resolution Sentinel-1. To assess FC-1’s time-series deformation monitoring capability, this study employed PSI and DSI techniques. The FC-1 satellite detected 241,696 PS points and 577,047 DS points, representing increases of 13.4-fold and 17.9-fold over Sentinel-1’s 18,076 PS points and 32,263 DS points, respectively. The average deformation velocity for FC-1’s DSI results was −0.13 mm/y, with its PSI results at −0.06 mm/y, compared to Sentinel-1’s rates of −0.13 mm/y and −0.43 mm/y, collectively indicating stability across the study area. Despite the differences in the density of the PS and DS points between the two satellites, both consistently identified deformation hotspots in areas R1 and R2, revealing localized subsidence and uplift near the stadium and the logistics park. Additionally, FC-1 identified further deformations in areas R3, R4, and R5, including in a construction material plant and farmland. In one farmland area, FC-1’s high-density PSs and DSs depicted a subsidence funnel with enhanced clarity. This underscores FC-1’s effectiveness in monitoring soft soil and vegetated areas with distributed scatterers under the DSI method. Over a complex overpass, using DSI with FC-1 achieved high-density PSs and DSs, reducing the dispersion of the deformation velocity points and clearly delineating the road section’s deformation velocity curve. Compared to Sentinel-1, FC-1 displayed lower dispersion in the deformation velocity points across both the DSI and PSI methods in this area. In terms of accuracy, FC-1’s data exhibited higher coherence and lower standard deviation in the deformation measurements than those of Sentinel-1. The mean error and root mean square error between FC-1’s DSI results and the leveling measurements were 1.761 mm and 2.172 mm, respectively, demonstrating FC-1’s millimeter-level accuracy in deformation monitoring. Moreover, the discrepancies between FC-1 and leveling measurements were smaller than those for Sentinel-1. The high-density PSs and DSs captured by FC-1, along with its high-precision time-series monitoring capabilities, underscore its suitability for detailed surface deformation analyses and health monitoring for infrastructure, especially in complex urban environments. A comparison between FC-1 and other high-resolution satellites, such as COSMO-SkyMed, will be addressed in a subsequent paper.

## Figures and Tables

**Table 1:** FC-1 and Sentinel-1 information.

Satellite Name	Number of Images/Scenes	Time Span	Orbit Direction	Angle of Incidence (°)	Revisit Cycle (Days)	Spatial Resolution
FC-1	20	12 October 2023–30 May 2024	Descending	38.8	11	3 × 3 m^2^
Sentinel-1	20	10 October 2023–6 June 2024	Descending	39.2	12	5 × 20 m^2^

**Table 2:** FC-1 and Sentinel-1 information.

Satellite Name	ADV	Select_Method	Percent_Rand	Unwrap_Time _Win	Unwrap_Grid_Size	Weed_Standard _Dev
FC-1	0.4	Percent	1	100	20	0.8
Sentinel-1	0.4	Percent	20	100	20	1.2

**Table 3:** Statistical table of time-series deformation differences for FC-1 and Sentinel-1.

Region	Average RMSE (mm)	Maximum Differences (mm)	Minimum Differences (mm)	Average Differences (mm)
Region A	0.802	6.417	0.0902	1.417
Region B	0.837	5.750	0.002	2.790
Region C	0.905	6.257	0.042	3.014
Region D	1.042	7.415	0.010	1.857

**Table 4:** Table of error statistics for FC-1 and Sentinel-1.

Satellite Name	RMSE (mm)	Maximum Error (mm)	Minimum Error (mm)	Average Error (mm)
FC-1	2.29	5.52	0.34	1.83
Sentinel-1	3.5	7.38	0.32	2.83

## Data Availability

The raw data supporting the conclusions of this article will be made available by the authors upon request.
